# Seroprevalence of *Toxoplasma gondii* in free-living European mouflon (*Ovis orientalis musimon*) hunted in central Germany

**DOI:** 10.1051/parasite/2018020

**Published:** 2018-04-10

**Authors:** Mike Heddergott, Natalia Osten-Sacken, Peter Steinbach, Alain C. Frantz

**Affiliations:** 1 Musée National d’Histoire Naturelle, 2160 Luxembourg Luxembourg; 2 Fondation Faune Flore, 2160 Luxembourg Luxembourg; 3 University of Göttingen, Faculty of Chemistry, 37077 Göttingen Germany

**Keywords:** *Toxoplasma gondii*, seroprevalence, mouflon, wildlife, MAT, central Germany

## Abstract

Despite increasing consumption of mouflon (*Ovis orientalis musimon*) meat in Germany, there is currently no surveillance of *Toxoplasma gondii* infection in populations of these animals and generally little knowledge about the prevalence of this protozoan in German wild ungulates. Between 2011 and 2015, we collected 138 blood samples from a free-living mouflon population in central German and tested sera for the presence of *T. gondii* antibodies using a modified agglutination test (MAT, cut-off 1:20). Antibodies were detected in 31 of the 138 samples (22.46%). There was a significant difference in seroprevalence between the different age classes, with antibodies to *T. gondii* more frequent in adults. In contrast, there was no significant difference in seroprevalence depending on sex and year of sample collection. Game meat is frequently consumed as raw or undercooked meat and may therefore represent a potential source of human infection with *T. gondii*.

## Abstract (fr)

Malgré une consommation en augmentation de viande de mouflon (*Ovis orientalis musimon*) en Allemagne, il n’existe actuellement aucune surveillance de l’infection par *Toxoplasma gondii* dans les populations de ces animaux et généralement peu de connaissances sur la prévalence de ce protozoaire chez les ongulés sauvages allemands. Entre 2011 et 2015, nous avons prélevé 138 échantillons de sang d’une population de mouflons vivant en liberté en Allemagne centrale et testé des sérums pour détecter la présence d’anticorps contre *T. gondii* en utilisant un test d’agglutination modifié (MAT, seuil de 1:20). Des anticorps ont été détectés dans 31 des 138 échantillons (22,46 %). Il y avait une différence significative de séroprévalence entre les différentes classes d’âge, avec des anticorps contre *T. gondii* plus fréquents chez les adultes. En revanche, il n’y avait pas de différence significative de séroprévalence selon le sexe et l’année de prélèvement des échantillons. Le gibier est fréquemment consommé sous forme de viande crue ou insuffisamment cuite et peut donc représenter une source potentielle d’infection humaine par *T. gondii*.

## Introduction

*Toxoplasma gondii* is a ubiquitous apicomplexan parasite of warm-blooded animals and humans [7]. Cats, which are the only known definitive host, shed unsporulated oocysts into environment [6]. Humans and wildlife can become infected by ingesting raw or undercooked meat with *T. gondii* tissue cysts or by consuming food or drink contaminated with oocysts [7,16]. *T. gondii* infection can be common in domesticated and wild animals intended for human consumption and the European Food Safety Authority (EFSA) has recommended the surveillance and monitoring of toxoplasmosis in humans, animals and foodstuffs [9].

The mouflon (*Ovis orientalis musimon*) is becoming an important game species in Germany. According to the German Hunting Federation (DJV), the annual hunting bag increased from around 6000 animals in the early 2000’s to around 8000 animals from 2015 onwards (www.jagdverband.de). Despite the concurrent increase in consumption of mouflon meat, there is currently no surveillance of *T. gondii* infection in populations of these animals and little knowledge about the prevalence of the protozoan in German wild ungulates generally [19,23].

Here, we aim to assess the seroprevalence of *T. gondii* in a free-living German population of mouflon, sampling carcases that were earmarked for human consumption.

## Material and methods

### Ethics

Mouflon are legally considered to be a game species in Germany and can be harvested by licensed hunters outside the closed season without special permission. No animals were killed with the aim of providing samples for this study. All hunted individuals were legally shot and made available to the authors.

### Sample collection

We sampled a small free-living mouflon population in a 190 km^2^ study site in western Thuringia, comprising the southern part of the Eichsfeld and the western part of the Unstrut-Hainich administrative districts. While the size of the population has not formally been established, estimates by local hunters range from 150 to 250 individuals. Mouflons were translocated from the eastern Harz Mountains to the Eichsfeld district during the late 1970’s [25], while further animals were illegally introduced to the Unstrut-Hainich district during the 1990’s. Between 2011 and 2015, we collected blood from the heart of 138 legally hunted mouflons, corresponding to 84% of all animals hunted in that period. Samples were centrifuged for 10 min at 1000 *g* using an EBA 200 (Hettich, Tuttlingen, Germany) and sera stored at −20 °C until analysis. We recorded the sex and age of each sampled animal as well as the year of collection. Based on horn size, males were classified as juveniles (≤ 1 year), yearlings (1-2 years) or adults (≥ 2 years), while females were similarly classified based on the dentition of the lower jaw [20,21].

### Determination of antibodies to *T. gondii*

Serum samples were tested for immunoglobulin G (IgG) antibodies against *T. gondii* with the modified agglutination test (MAT) using a commercial kit (Toxo-Screen DA^®^, bioMérieux, Lyon, France). Positive and negative controls were based on formalin-fixed tachyzoites as antigens. Each serum sample was tested at dilutions of 1:20, 1:400, 1:1600 and 1:3200. A cut-off titer of 1:20 was chosen to maximize both sensitivity and specificity of the test [8]. Among all the serological tests available, the MAT has been used extensively for the diagnosis of toxoplasmosis in both domestic and wildlife species because it is considered to be the most reliable to detect antibodies to *T. gondii* in animals, especially in latently infected animals [7].

### Statistical analysis

The effect of sex, age class, and collection year on *T. gondii* seroprevalence was analyzed using a *χ*^2^ test performed in SPSS v.22 (SPSS Inc., Chicago, Illinois, USA). A *p-*value of < 0.05 was chosen as the cut-off point for statistical significance. Odds ratios (ORs) and their 95% confidence intervals (95% Cls) were estimated to explore the strength of the association between the presence of *T. gondii* positivity and the explanatory variables.

## Results

Antibodies to *T. gondii* were detected in 31 of the 138 (22.46%, 95% CI: 15.41-29.51) analyzed mouflons (Table 1). Positive results were recorded at titers between 1:20 (32.26%), 1:400 (51.61%), and 1:1600 (16.13%). While seroprevalence was higher in males (19/76; 25%) than females (12/62; 19.35%), this difference was not significant (*p* = 0.429). Similarly, there was no significant difference in seroprevalence between collection years (*p* = 0.967). In contrast, there was a significant difference in seroprevalence between the different age classes (*p* = 0.002), with antibodies to *T. gondii* frequently present in adults in particular (Table 1).

**Table 1:** Seroprevalence of *Toxoplasma gondii* in mouflon by sex, age and collection year.

Variable	Category	No. tested	No. positive	Prevalence in % (95% CI)	*p*-value	OR (95% CI)
Sex	Male	76	19	25.00 (15.04-34.96)	0.429	Reference
	Female	62	12	19.35 (9.24-29.47)		0.72 (0.32-1.63)
Age	≤ 1 year	36	5	13.89 (2.02-25.76)	0.002	Reference
	1-2 year	61	9	14.75 (5.60-23.91)		1.07 (0.33-3.49)
	≥ 2 year	41	17	41.46 (25.72-57.21)		4.39 (1.42-13.60)
Collection year	2011	16	4	25.00 (1.17-48.83)	0.967	Reference
	2012	31	6	19.35 (4.62-34.09)		0.72 (0.17-3.04)
	2013	37	9	24.32 (9.82-38.83)		0.96 (0.25-3.75)
	2014	28	7	25.00 (7.90-42.10)		1.00 (0.24-4.13)
	2015	26	5	19.23 (3.00-35.46)		0.71 (0.16-3.18)
Total		138	31	22.46 (15.41-29.51)		

## Discussion

In the present study, we observed a relatively high seroprevalence of *T. gondii* (22.46%) in a free-living German mouflon population. A similarly high value (24.4%) was reported from captive Czech mouflons (based on an enzyme-linked immunosorbent assay, ELISA) [18]. Other studies that were also based on the MAT test reported lower prevalence values < 15% [1,2,10], while studies using different techniques for antibody detection reported even lower values < 10% [3,13].

The fact that we observed a relatively high frequency of *T. gondii* antibodies in a free-living herbivore suggests relatively high environmental contamination with oocysts. Rural habitats (forest, meadows, agricultural areas) like our study area can be exploited by the European wildcat (*Felis* *silvestris* *silvestris*) and/or feral domestic cats (*Felis silvestris catus*) [5,17], both of which have been reported to have a high serological prevalence of *T. gondii* antibodies in Germany [11,22,24]. Our study area is indeed located within the core distribution area of the wildcat in central Germany [14]. Altogether, 38.3% of raccoons (*Procyon lotor*) sampled in the same study area were recently shown with a MAT test to be serologically positive for the protozoan [12].

Our results suggest that mouflons older than two years of age had a higher seroprevalence than younger animals, while there was no significant difference in seroprevalence depending on sex and year of sample collection. Infection with *T. gondii* is frequently more prevalent in adult than juvenile animals, since the cumulative likelihood of exposure to *T. gondii* increases with age and the antibodies have a lifelong persistence [1,15]. Since we did not observe a significant difference in seroprevalence between years, our results suggest that the environmental contamination with infective oocysts remained constant throughout the study. Almería *et al.* [1] found a significant difference in seroprevalence of *T. gondii* in wild ruminants in Spain between hunting seasons and explained this result by some study years being exceptionally dry, hindering the persistence of infective oocysts. In our case, it is likely that the rate of oocyst shedding from feral cats and wildcats remained constant, as did the environmental conditions during the study. Finally, as male and female mouflons live permanently in mixed herds, a difference in exposure risks between the two sexes is unlikely.

Concurrent with an increase in the number of hunted animals, there has been an increase in the consumption of mouflon meat in Germany. In many parts of Germany, including our study area, hunters produce home-made sausages derived from raw or undercooked meat, leading to a risk of food-borne transmission of *T. gondii*. Verma *et al.* [26] isolated viable *T. gondii* from two mouflons that were seropositive for the parasite and Calero-Bernal *et al.* [4] detected the presence of *T. gondii* with a polymerase chain reaction in 3 out of 12 tested mouflon (25%). Further studies on different free-ranging mouflon populations are therefore required to assess infection levels in meat and the derived products intended for human consumption, and to assess the risk of transmission of the pathogen to humans.

## Conclusions

This is the first epidemiological report of *T. gondii* prevalence in free-living mouflons in Germany. We show that *T. gondii* infection is prevalent in mouflons of all age ranges in a central German study population. This may represent a potential source of human infection with *T. gondii*.

## Conflict of interest

The authors declare that they have no conflicts of interest in relation to this article.
